# Characterizing *Staphylococcus aureus* genomic epidemiology with multilevel genome typing

**DOI:** 10.1128/msystems.00935-25

**Published:** 2025-10-02

**Authors:** Michael Payne, Liam Cheney, Sandeep Kaur, Genevieve McKew, Ruiting Lan

**Affiliations:** 1 School of Biotechnology and Biomolecular Sciences, University of New South Wales 98492, Sydney, New South Wales, Australia; 2 Department of Microbiology and Infectious Diseases, Concord Repatriation and General Hospital, Sydney, New South Wales, Australia; 3 Concord Clinical School, Faculty of Medicine and Health, University of Sydney 522555, Sydney, New South Wales, Australia; LifeMine Therapeutics, Cambridge, Massachusetts, USA

**Keywords:** *Staphylococcus aureus*, multilevel genome typing, epidemiology, genomics, genomic nomenclature, public health surveillance, database

## Abstract

*Staphylococcus aureus* is a major source of both hospital- and community-acquired infections worldwide. Advances in whole-genome sequencing (WGS) technologies have recently generated large volumes of *S. aureus* WGS data. The timely classification of *S. aureus* WGS data using genomic typing technologies has the potential to describe detailed genomic epidemiology at large and small scales. In this study, a multilevel genome typing (MGT) scheme, consisting of eight levels of multilocus sequence typing (MLST) schemes of increasing resolution, was developed for *S. aureus* and was used to analyze 50,481 publicly available genomes. The application of MGT to *S. aureus* epidemiology was shown in three case studies. First, the population structure of the globally disseminated MLST sequence type 8 (ST8) was described by MGT2 and compared with *Spa* typing. Second, MGT was used to characterize MLST ST8-USA300 isolates that colonized multiple body sites in the same patient. Finally, the MGT was used to describe the transmission of MLST ST239-SCC*mec* III throughout a single hospital. MGT STs were able to describe both isolates that had spread between wards and those that had colonized different reservoirs within a ward. *S. aureus* MGT describes *S. aureus* genomic epidemiology at multiple resolutions ranging from the global spread to local/individual scale using stable and standardized ST assignments. The *S. aureus* MGT database (https://mgtdb.unsw.edu.au/staphylococcus) is capable of tracking new and existing clones to facilitate the design of new strategies to reduce the global health burden of *S. aureus* infections.

**IMPORTANCE:**

*Staphylococcus aureus* causes both hospital- and community-acquired infections worldwide. Methicillin-resistant *S. aureus* is best known and has spread across the globe. Whole-genome sequencing (WGS) can type strains at the highest resolution. To enable best use of WGS data for surveillance of *S. aureus*, this study developed a multilevel genome typing (MGT) scheme that provides a publicly available, standardized, flexible, and easily communicated system to describe *S. aureus* strains. MGT has eight typing levels that provide progressively higher resolution. Each of these levels allows subtypes to be accurately identified and tracked. We show that MGT can be used to track well-known *S. aureus* strains at low resolution while simultaneously being able to track outbreaks in hospital settings at high resolution. The *S. aureus* MGT will facilitate the use of genomic data for surveillance without the need for bioinformatic expertise, improving efforts to control this important pathogen and prevent infections.

## INTRODUCTION

*Staphylococcus aureus* causes infections in both hospitalized individuals and those without clinical associations who are otherwise considered “healthy” (1). In 2017, *S. aureus* caused over 120,000 bloodstream infections and 20,000 deaths in the USA alone (2). A hallmark of *S. aureus* is its ability to acquire new mechanisms of antimicrobial resistance (AMR). The first report of methicillin-resistant *S. aureus* (MRSA) was in 1961, followed by a series of epidemic waves, wherein each acquired additional AMR (3). The spread of these epidemic waves was predominantly reported in individuals associated with hospitals, and the isolates were referred to as hospital-associated MRSA (HA-MRSA) (4). In the 1990s, there were reports of individuals not associated with clinical settings carrying MRSA. These cases were defined as community-associated MRSA (CA-MRSA). Recent epidemiological surveillance has reported the spread of CA-MRSA into hospital settings, with CA-MRSA predicted to displace HA-MRSA in most countries as the leading cause of MRSA infections (5, 6).

Early applications of multilocus sequence typing (MLST) retrospectively studied the global spread of HA-MRSA. One such example is sequence type 239 (ST239), which spread globally and caused multiple epidemics of HA-MRSA (7). MLST has also been used to characterize the emergence and spread of CA-MRSA. A comparative study between HA-MRSA and CA-MRSA has shown that CA-MRSA is more diverse in sequence types (STs) and geographically restricted (8). MLST is informative for broad epidemiological resolution analysis (9), providing utility in studying the origin and evolution of *S. aureus* (8). Other frequently used broad-resolution typing methods, commonly used in conjunction with MLST, include typing of the hypervariable Staphylococcal protein A (*spa* typing) and staphylococcal cassette chromosome *mec* typing (SCC*mec* typing) (8, 10, 11). SCC*mec* is a mobile genetic element and a determinant for broad-spectrum β-lactam resistance. SCC*mec* types are commonly used in conjunction with MLST types. So far, 13 different types of SCC*mec* elements have been discovered, which are further divided into subtypes based on the differences in their joining regions (8). SCC*mec* gene variants are discovered regularly. *S. aureus* strains are universally characterized using both MLST and SCC*mec* types. *Spa* typing, on the other hand, is often used for local or hospital outbreaks. The main source of variation in *spa* types is alterations (such as duplications, deletions, or mutations) in the repeat units of the polymorphic X region of the gene. Within closely related strains, *spa* types remain relatively stable. However, strain lineages cannot be reconstructed by direct sequence comparisons based on duplications/deletions of repeats (8). Furthermore, occasionally, recombination and/or homoplasy can lead to misclassification of types (12).

*S. aureus* whole-genome sequencing (WGS) data, however, are most often analyzed through phylogenomic reconstruction, especially when high-resolution strain differentiation of strains is required. Comparison of geographically diverse isolates sampled over long periods has uncovered the origins of globally disseminated clones (13–16). For example, a study of ST8 isolates sampled across the globe identified its European origin and transmission into North America in the 2000s (13). Once ST8 was established in North America, phylogenetic analyses confirmed subsequent parallel epidemics of ST8-USA300 and ST8-USA400 CA-MRSA in North and South America, respectively (17).

While investigating *S. aureus* genomic epidemiology using phylogenetic approaches has provided high-resolution comparisons, phylogenetics without further implementation of genotyping algorithms cannot easily assign standardized names to strains (i.e., classify isolates into “types”). In 2014, an *S. aureus* core genome MLST (cgMLST) scheme was developed that offered high-resolution typing and standardized nomenclature (18). cgMLST has been sporadically used in small-scale studies, such as outbreaks in neonatal wards and household transmission (19–21). cgMLST for each of these investigations distinguished epidemiologically indistinguishable isolates and allowed clustering of cgMLST sequence types (cgSTs) to describe the population structure. However, cgMLST for species-wide classification is hampered by the lack of flexibility in typing resolution. Existing cgMLST applications require setting allele thresholds to cluster isolates. Selecting different allele thresholds between investigations would prevent establishing a standardized nomenclature; to date, no such allele thresholds have been rigorously investigated and defined. Furthermore, the ability to vary the typing resolution is essential for describing large-scale epidemiology, such as the global dissemination of ST8, and small-scale epidemiology, such as ST8-USA300 outbreaks within a hospital setting (13, 20).

**Fig 1:**
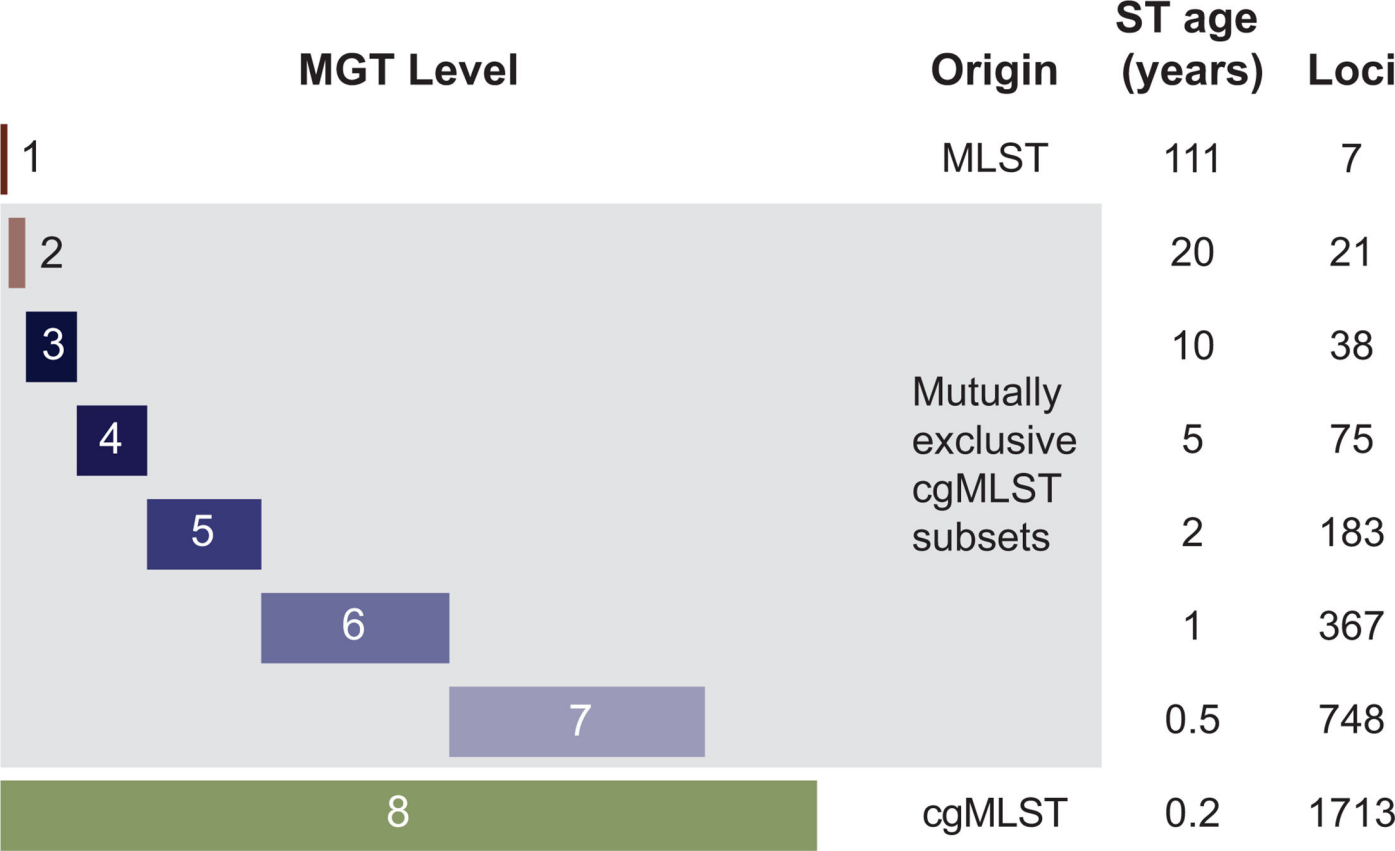
Schematics of the MGT system. The *S. aureus* MGT scheme, as shown, consisted of eight levels with increasing resolution. The lowest-resolution level, MGT1, is the classic *S. aureus* MLST scheme, while the highest-resolution level, MGT8, is the species core genome MLST scheme. Levels 2 to 7 are composed of a mutually exclusive subsets of cgMLST loci. The age of an ST is defined as the average time for a new allele to emerge to give rise to a new ST at a given MGT level.

We previously developed a new method called multilevel genome typing (MGT) that has been applied to several organisms (22–25). The MGT comprises a series of MLST schemes of increasing sizes, thus providing higher resolutions with each higher level. The advantage of using a series of MLST schemes within a single system is that at each level, an ST is assigned, which is a stable “type” assigned to an isolate without relying on grouping isolates based on multiple allele thresholds, which inherently lack this stability. For a species, MGT is typically organized such that MGT1 is the same as traditional seven-gene MLST, while MGT8 is the species cgMLST. MGT2 to MGT7 have an increasing number of loci as shown in Fig. 1 schematically. The lower-resolution levels can be used for longer-term epidemiology, while the higher-resolution levels can be used for shorter-term epidemiology. Following the well-established MLST terminology, an ST is assigned at each MGT level. Therefore, a strain can be typed at each level with specific STs at a given level, or the STs can be concatenated and referred to as a genome type, providing a standardized nomenclature for epidemiological typing.

This study developed an *S. aureus* MGT that (i) offered flexibility in typing resolution to describe large- and small-scale *S. aureus* genomic epidemiology and (ii) established a standardized nomenclature for unambiguous communication of *S. aureus* types between investigations.

## MATERIALS AND METHODS

### Data set curation

Paired-end short-read data sets for 66,238 genomes that were sequenced using the Illumina Genome Analyzer, HiSeq, MiSeq, NextSeq, and NovaSeq platforms were downloaded on 15 July 2019 from the Sequence Read Archive of the National Center for Biotechnology Information (NBCI). Additionally, publicly available “year of isolation” metadata, where available, were downloaded from NCBI BioSample. Read sets were screened for contamination with Kraken (v.1.0.0), and read sets with more than 20% non-*S*. *aureus* reads were removed (26). Assemblies were generated with the MGTdb pipeline, which trimmed reads with Trimmomatic (v.0.39.0), performed reference-based assembly with Shovill (v.1.0.9) and SKESA (v.2.3.0), and calculated assembly quality metrics with Quast (v.5.2.0) (27–30). The assemblies were quality-filtered using the thresholds shown in Table S1. The quality-filtered species data set contained 50,481 genomes (Table S2).

### Core genome validation

A data set representative of all diversity within *S. aureus* was selected, which comprised assemblies from each MLST ST. This data set was termed the representative data set. The number of assemblies per ST included in the representative data set was proportionate to the frequency of that ST. All assemblies assigned a singleton ST were included.

A *S. aureus* core genome was previously defined with 1,861 core loci (18). The core loci of this core genome were verified using the representative data set (Supplementary Methods 1.7). We used two metrics to measure the quality of each locus: first, the number of assemblies of the representative data set in which locus presence was present (or absent), and second, the percentage of assemblies in which additional processing was required for a locus to be called (problematic locus count). Additional processing scripts handled loci with missing sequence. A locus was reported as absent when greater than 20% of the sequence was missing and problematic when at least 80% of the DNA sequence was present (22). Core loci that were either absent or problematic in more than 1% of the representative data set were removed. The validated core genome had 1,713 core genes.

### MGT design

MGT1 loci were the same as the seven-gene MLST for *S. aureus* (31). MGT8 loci were the validated core genome genes. The MGT2 to MGT7 loci were selected from the validated core genome. MGT2–MGT7 loci were selected based on previously published methods (22–24). The MGT design methodology included four stages: calculating the size of each level, calculating the selection criteria for each core locus, separating the core loci into preferences, and selecting core loci to fill the levels (Supplementary Methods, Supplementary Data sets, Supplementary Scripts). Loci within MGT levels 2–7 were mutually exclusive, allowing the independent assignment of strain relationships. This independence leads to hierarchical inconsistency, defined as strains assigned to the same ST at a higher level of resolution but split into multiple STs at a lower-resolution level, which occurs for a small proportion of the strains (see Supplementary Methods 1.6).

### MGT typing of the *S. aureus* species data set

The species data set (*n* = 50,481) was processed using the MGT pipeline (available on GitHub at: https://github.com/LanLab/MGT_reads2alleles) (22). First, as MGT1 is the well-established seven-gene MLST for *S. aureus* (31), all MGT1 STs are identical to the MLST STs, and hence their assignment is made using the mlst (https://github.com/tseemann/mlst) program with the *S. aureus* database hosted on PubMLST (32). The MGT2 to MGT8 STs are assigned using the MGT allele-calling pipeline. Core locus reference alleles were selected using the available complete reference genome *S. aureus* subsp. *aureus* COL (NCBI SRA accession: GCF_000012045). Both the reference alleles and their corresponding MGT STs, based on the allelic profiles for each MGT level, were initially assigned the integer identifier “1.” For subsequent isolates, the MGT allele-calling pipeline applied the following thresholds: a maximum of 16 single nucleotide polymorphisms (SNPs) within a 40-base sliding window, 80% BLAST nucleotide identity, and 80% BLAST high-scoring segment pair. The 16 SNPs within a 40-base sliding window were implemented to account for potential misalignments during allele calling. New alleles identified for any locus were assigned the next available integer identifier. Similarly, newly observed allelic profiles at each MGT level were assigned the next available integer for their corresponding MGT ST. These integer identifiers are arbitrary, i.e. differences between the identifiers do not imply genetic relatedness between isolates.

### Allele-based phylogenetic construction

Allele-based phylogenies were constructed for MGT1 ST8 isolates. Allele profiles from MGT8 (equivalent to cgMLST) were used to generate a phylogenetic tree using GrapeTree (v.1.0.0) with the rapid neighbor-joining algorithm (33). The MLST ST8 phylogeny was compared with the MGT classification of the MLST ST8 isolates. The isolates in the phylogeny generated by GrapeTree are colored by their assigned MGT2 ST.

### SNP-based phylogenetic construction

Two SNP-based phylogenies were generated, one each for populations of ST239-SCC*mec* III and ST8-USA300 (34, 35). Except for the chosen reference genome, SNP-based phylogenies were generated for each population using the same process. The reference genome *S. aureus* JKD6008 (NCBI SRA accession: GCA_000145595) was used to generate the ST239-SCC*mec* III phylogeny, and the reference genome *S. aureus* TCH1516 (NCBI SRA accession: GCF_000017085) was used to generate the ST8-USA300 phylogeny. To generate both phylogenies, SNPs were called against the reference genome and an SNP alignment was generated using default Snippy-Core settings (v.4.6.0) (36). SNPs under the influence of recombination were predicted and removed from SNP alignment using RecDetect (v.6.1) (37). RecDetect predicted recombination SNPs using a strict recombination prediction model. IQ-TREE (v.2.0.4) processed the SNP alignment to create a maximum likelihood phylogeny using 1,000 bootstraps and automatic selection of model parameters (38). Additional metadata were visualized on the phylogeny using iTOL (v.6) (39).

### *In silico Spa* and SCC*mec* typing

All *spa* type comparisons used *spa* types predicted *in silico* by SpaTyper (v.0.3.3) with default settings (40). SCC*mec* types were predicted using staphopia-sccmec (v.1.0.0) with default settings (41). It should be noted that the publicly available version of Staphopia used in this study can only identify SCC*mec* types I–VII, and recently described types (VIII–XIV) were not identifiable (42).

## RESULTS

### The *S. aureus* MGT consists of eight levels

*S. aureus* MGT consisted of eight levels with increasing numbers of loci (Fig. 1; Table 2). MGT1 was the traditional seven-gene *S. aureus* MLST scheme, and MGT8 was the species cgMLST, which had 1,713 core loci. The intermediate MGT levels of MGT2, 3, 4, 5, 6, and 7 each had 21, 38, 75, 183, 367, and 748 loci, respectively (Fig. 1; Table 2; Data set S9). From the global data set (*n =* 50,481), 98.73% (49,838/50,481) of the isolates were assigned an ST at all MGT levels. The remaining 1.27% (643/50,481) of the isolates were not assigned an ST at one of the eight MGT levels.

### Epidemiology of the *S. aureus* species described using MGT

The division of the *S. aureus* population structure by MGT was examined using the species data set (50,481 isolates; Table S2). Of this data set, 26,416 (52.3%) had year metadata, 27,046 (53.6%) had country metadata, and 25,596 (50.7%) had both metadata types. The average number of years that major STs were sampled showed a clear trend of shorter timespan in higher-resolution MGT levels (Table 1; Fig. S1), demonstrating the potential epidemiological usefulness of each level in describing short- and long-lived clones.

**TABLE 1:** Overview of the *S. aureus* MGT levels

MGT level	No. of loci	No. of major STs^*a*^	Isolates in major STs	% of isolates in major STs	Average year span of major STs	% of isolates in continent-specific major STs^*b*^
MGT1	7	144	46,621	96.14	17.29	30.22
MGT2	21	338	33,983	70.95	11.36	50.22
MGT3	38	450	26,234	54.78	8.6	84.27
MGT4	75	429	17,788	37.15	6.84	92.98
MGT5	183	339	9,721	20.30	4.07	96.48
MGT6	367	243	6,265	13.08	1.75	96.17
MGT7	748	173	4,095	8.55	1.07	99.4
MGT8	1,713	113	2,436	5.08	0.3	100

^*a*^ Major STs, STs with >10 isolates assigned to them.

^*b*^ Continent-specific STs, >80% of the assigned isolates from one continent.

**Fig 2:**
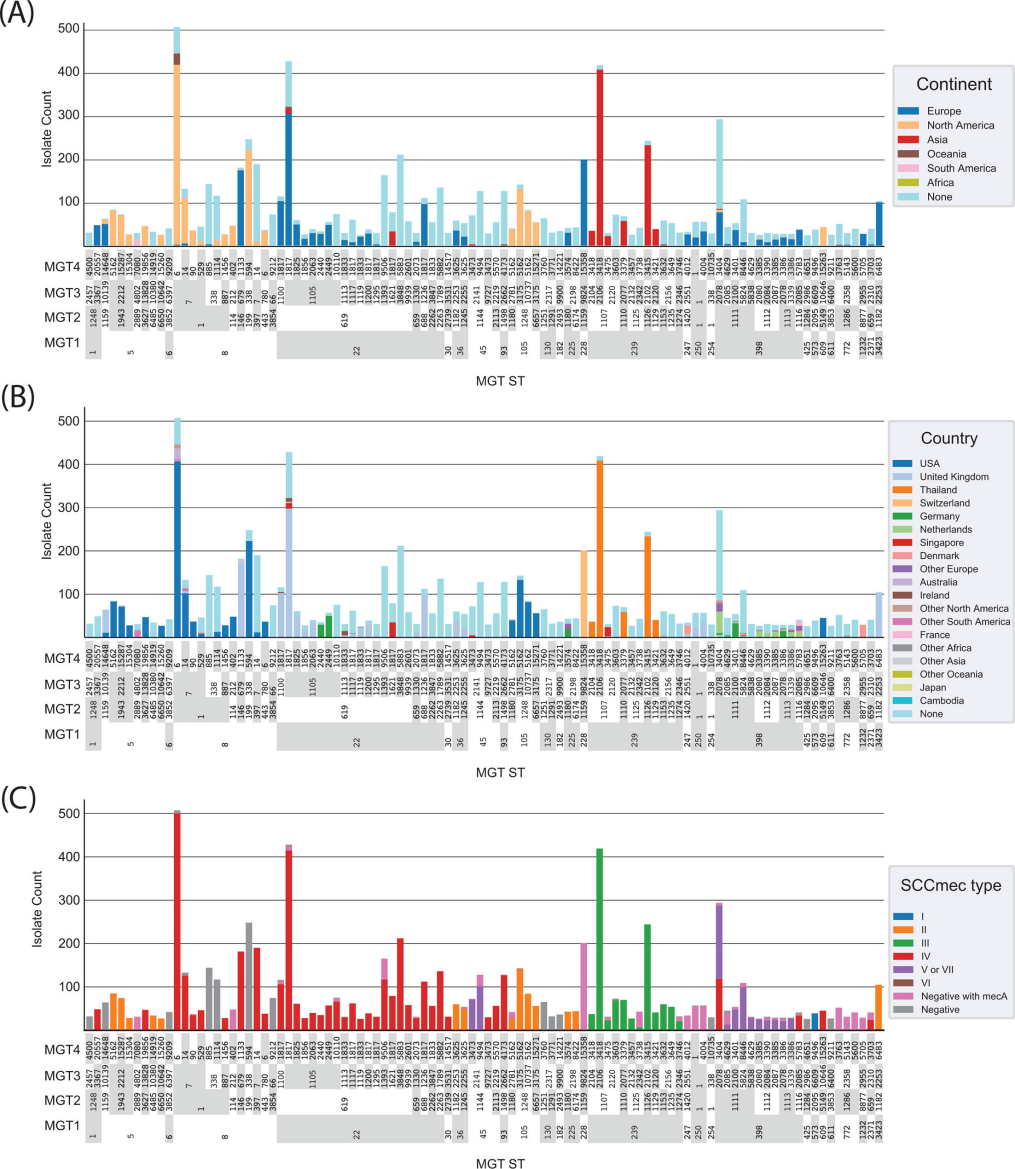
Geographic and genetic diversity of *S. aureus* as described by MGT. The figure plots the 100 largest MGT4 STs with continent and country metadata in panels A and B and carriage of *mecA,* and *mecC* in panel C to show MSSA and MRSA. The *y*-axis is isolate count in each ST. MGT1, MGT2, and MGT3 STs are shown below each MGT4 ST and grouped to allow examination of STs at these levels. (**A**) The proportion by continent in each MGT4 ST is represented by column colors. (**B**) The proportion by country in each MGT4 ST is represented by column colors. Countries with fewer than 200 isolates were grouped into “other continent” categories for clarity. (**C**) SCC*mec* types assigned in each MGT4 ST. Several instances of hierarchical inconsistency can be observed in this figure. This occurs when one ST at a higher resolution (i.e., MGT3 ST338) is found in multiple STs at lower resolutions (i.e., MGT2 ST1 and ST199). This is an expected outcome of the MGT scheme design used in this study (see Supplementary Methods 1.6).

To determine the level that would best describe geographical trends, we identified major STs (STs with more than 10 isolates assigned to them) with over 80% of their isolates from one continent. We then identified the lowest-resolution level, where more than 90% of the isolates were within these continent-specific STs (Table 1). The value of MGT4 was 92.98%; therefore, this level was selected to examine the distribution of continent-specific STs. The 100 largest MGT4 STs contained 7,952 isolates, 4,343 of which included continental metadata (Fig. 2A). These STs showed distinct distributions in both continent and country (Fig. 2B). Of the largest 100 STs, 26 contained no continent metadata, and the remaining 74 were continent specific. Of these, 63 were specific to one country, whereas 11 were found in more than one country on the same continent.

SCC*mec* types were also assigned to all isolates, and their distribution among the top 100 MGT4 STs is shown (Fig. 2C). In many cases, all isolates within an MGT1 ST were MRSA (or at least contained *mecA*) and were of a single SCC*mec* type (e.g., MGT1 ST22 and SCC*mec* IV). In other cases, MGT2 or MGT3 STs were required to describe groups of isolates that were either exclusively methicillin-sensitive *S. aureus* (MSSA) or MRSA and contained a single SCC*mec* type (e.g., MGT3 ST7: type IV, MGT3 ST338: MSSA, and MGT3 ST2141: type “V or VII”). At MGT4, only ST 3404 contained two SCC*mec* types (IV and “V or VII”).

Penicillin-sensitive *S. aureus* (PSSA), which lacks both *blaZ* and *mecA/mecC* and lacks resistance to penicillin, has recently emerged as a growing cause of infection (43, 44). We identified 9,318 (18.5% of the total data set) putative PSSA isolates (lacking *blaZ, mecA,* and *mecC*) and identified PSSA STs in the 100 largest MGT4 STs as described above. Six MGT4 STs were putative PSSA and were classified into four MGT1 STs (Table 2). These MGT1 STs were assigned to two clonal complexes (CCs) (ST5 and ST6 to CC5 and ST8 and ST254 to CC8).

**TABLE 2:** PSSA MGT4 STs in top 100 largest STs

Clonal complex	MGT1 ST	MGT4 ST	Isolates
5	5	14,648	68
	6	9,209	53
8	8	594	230
		885	141
		1,114	125
	254	10,735	30

### Using higher-level MGT to describe MGT1 ST8 isolates

We selected MGT1 ST8 (traditional MLST ST8, a well-known CA-MRSA clone) to demonstrate the application of MGT in *S. aureus* epidemiology. Of the 50,481 isolates typed, 4,388 were MGT1 ST8 and had associated collection year metadata. As the MGT levels increased in resolution, the number of STs at each level increased, while the size of STs decreased (Fig. S2). The largest MGT2 ST (ST1) was assigned to 37.58% (1,649/4,388) of the isolates, whereas the largest MGT5 ST (ST900) was assigned to 5.38% (236/4,388).

**Fig 3:**
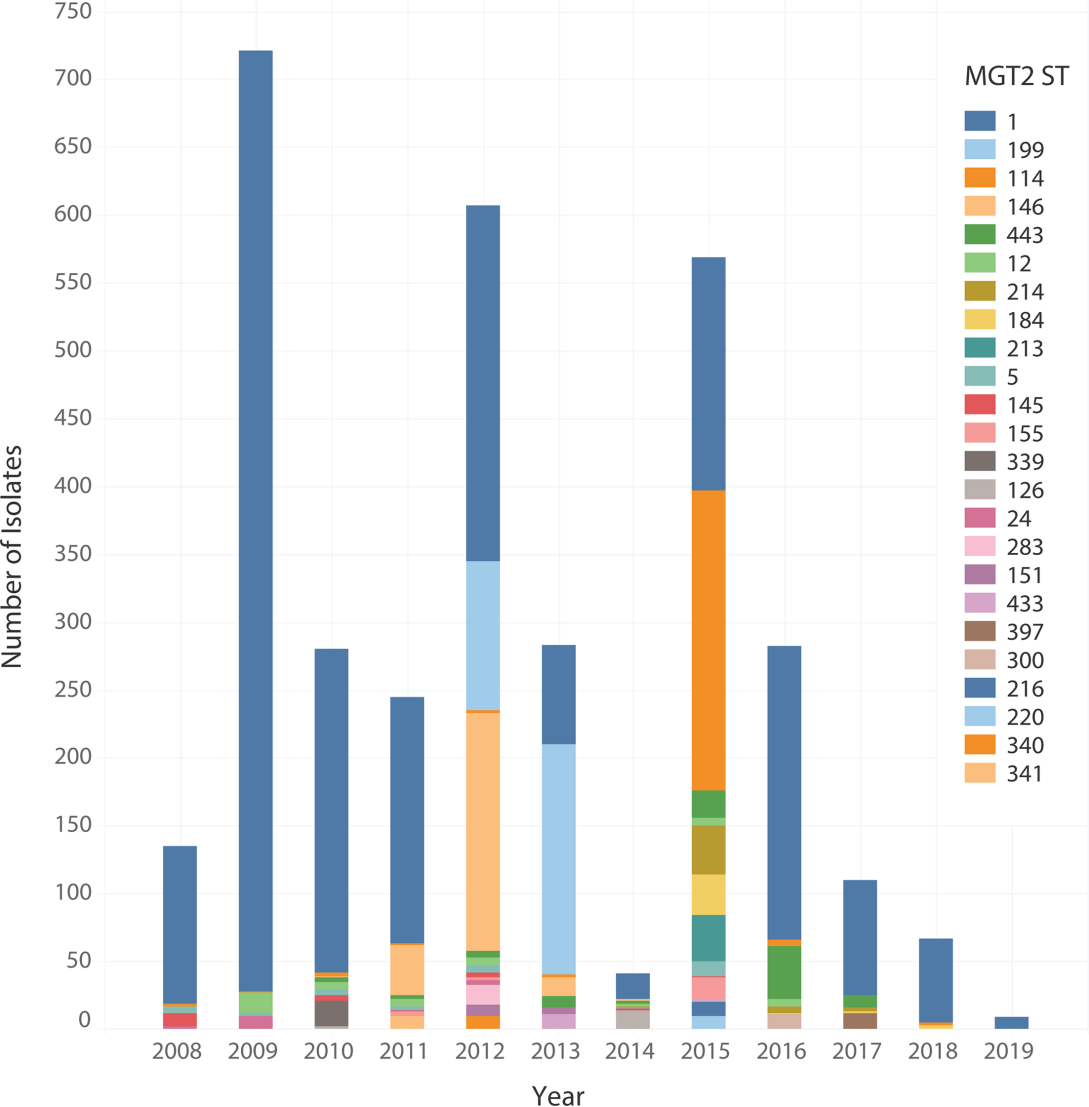
Distribution of MGT1 ST8 isolates by year and MGT2 STs. The temporal distribution of 3,478 MLST ST8 isolates colored by MGT2 ST. The size of each bar represents the number of isolates assigned to each MGT2 ST. MGT2 STs in the figure legend are organized in descending order of frequency.

In MGT2, there were 24 STs with >10 isolates and these isolates were sampled from 2008 to 2019. These 24 MGT2 STs varied in frequency over time (Fig. 3) with some STs persisting over multiple years and others sampled only in a single year. MGT2 ST1 was the only MGT2 ST sampled in all years and was by far the largest. In eight of these years (2008–2011 and 2016–2019), MGT2 ST1 was assigned to more than 70% of the isolates. The second-largest type, MGT2 ST199, was sampled only in 2012 and 2013. The third largest ST, MGT2 ST114, was similar to MGT2 ST1 and was sampled in all years except for 2014 and 2017. There was a considerable difference between MGT2 ST114 and MGT2 ST1 based on the frequency of isolates over time. MGT2 ST114 had 45.17% (262/580) of isolates sampled in 2015, and an average of 2.25 isolates for the remaining eight years (2008–2013, 2016, and 2018).

**Fig 4:**
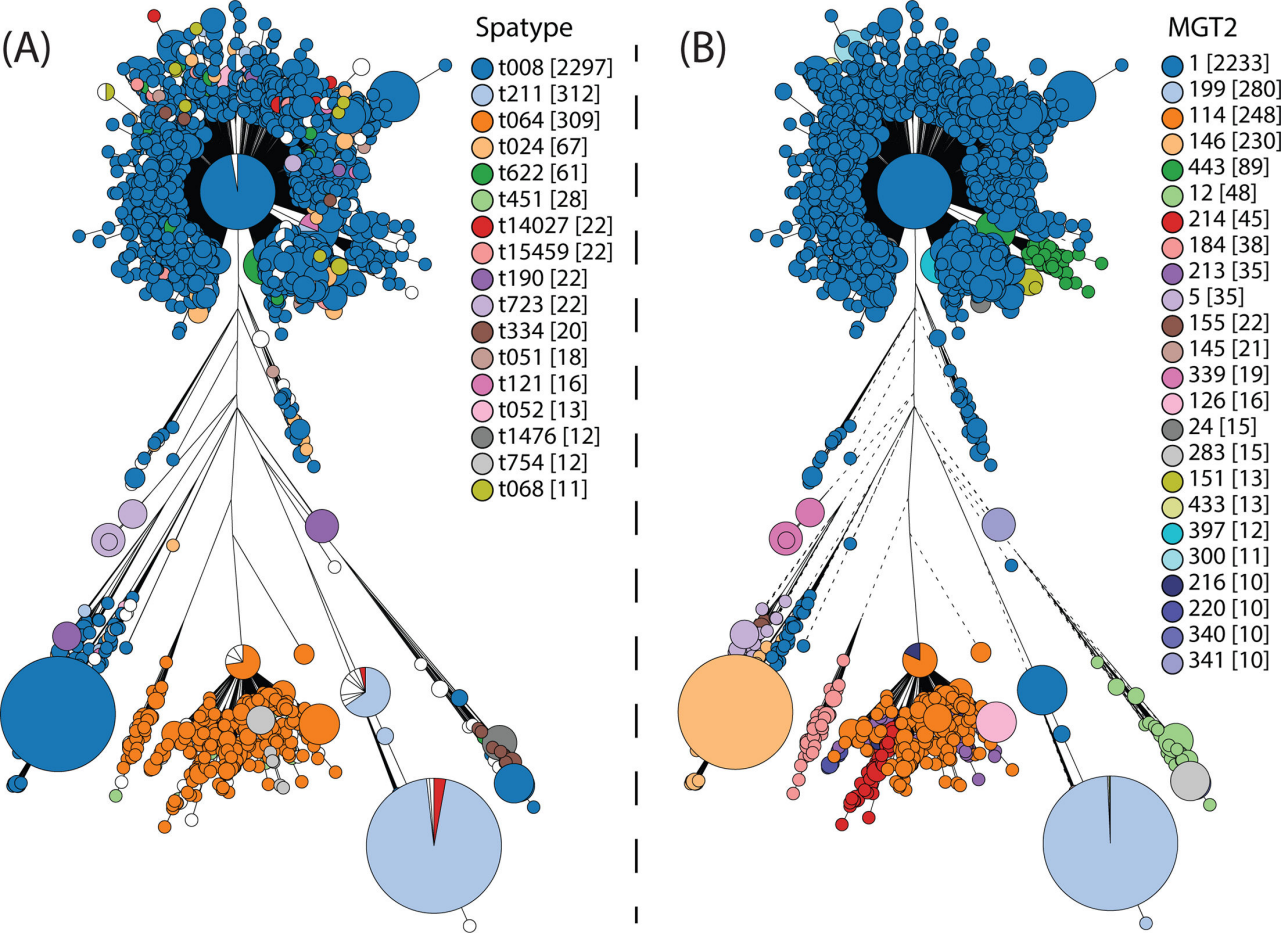
Comparing *spa* and MGT2 typing within MGT1 ST8. The MGT1 ST8 phylogeny (*n =* 3,488) was compared with that of the MGT2 and *spa* typing. The phylogeny was generated with GrapeTree (v.1.0.0), which uses the rapid neighbor-joining algorithm to process MGT8 (cgMLST) allele profiles (33). Each node was an MGT8 ST, and the nodes were colored by *spa* type (**A**) and MGT2 STs (**B**). Only *spa* types and MGT2 STs assigned to 10 or more isolates are shown. The frequencies of each type are shown in square brackets.

MGT typing was compared with *spa* typing of MGT1 ST8 isolates. The *spa* types of the MGT1 ST8 isolates were predicted *in silico*. A *spa* type was predicted in 99.91% (3,475/3,478) of the isolates, and there were 121 unique *spa* types (Fig. 4A). A large number of predicted *spa* types were small. Of the 121 *spa* types, 104 were assigned to fewer than 10 isolates, which cumulatively represented 6.15% (214/3,478) of the MGT1 ST8 data set. The majority of the 104 *spa* types (54.81%, 57/104) were assigned to a single isolate (Table S2).

The *spa* types assigned to 10 or more isolates were selected for comparison with MGT2 STs. Seventeen *spa* types were selected that together typed 93.85% (3,264/3,478) of the MGT1 ST8 isolates (Fig. 4B). The majority of the isolates were assigned to *spa* type t008 (66.04%, 2,297/2,478). The next two largest *spa* types were t211 and t064, which included 312 and 309 isolates, respectively. The remaining 14 *spa* types were assigned to fewer than 100 isolates.

The division of the MGT1 ST8 data set into MGT2 STs and *spa* types was compared (Fig. 4; Fig. S3). Eight MGT2 STs had a single *spa* type. MGT2 ST283, ST300, and ST340 isolates were *spa* type t008, MGT2 ST126 and ST216 were *spa* type t064, MGT2 ST341 was *spa* type t190, MGT2 ST339 was *spa* type t723, and MGT2 ST397 was *spa* type t622. Other MGT2 STs had a predominant *spa* type with one or more other *spa* types.

### MGT investigation of MGT1 ST8 asymptomatic *S. aureus* colonization

**Fig 5:**
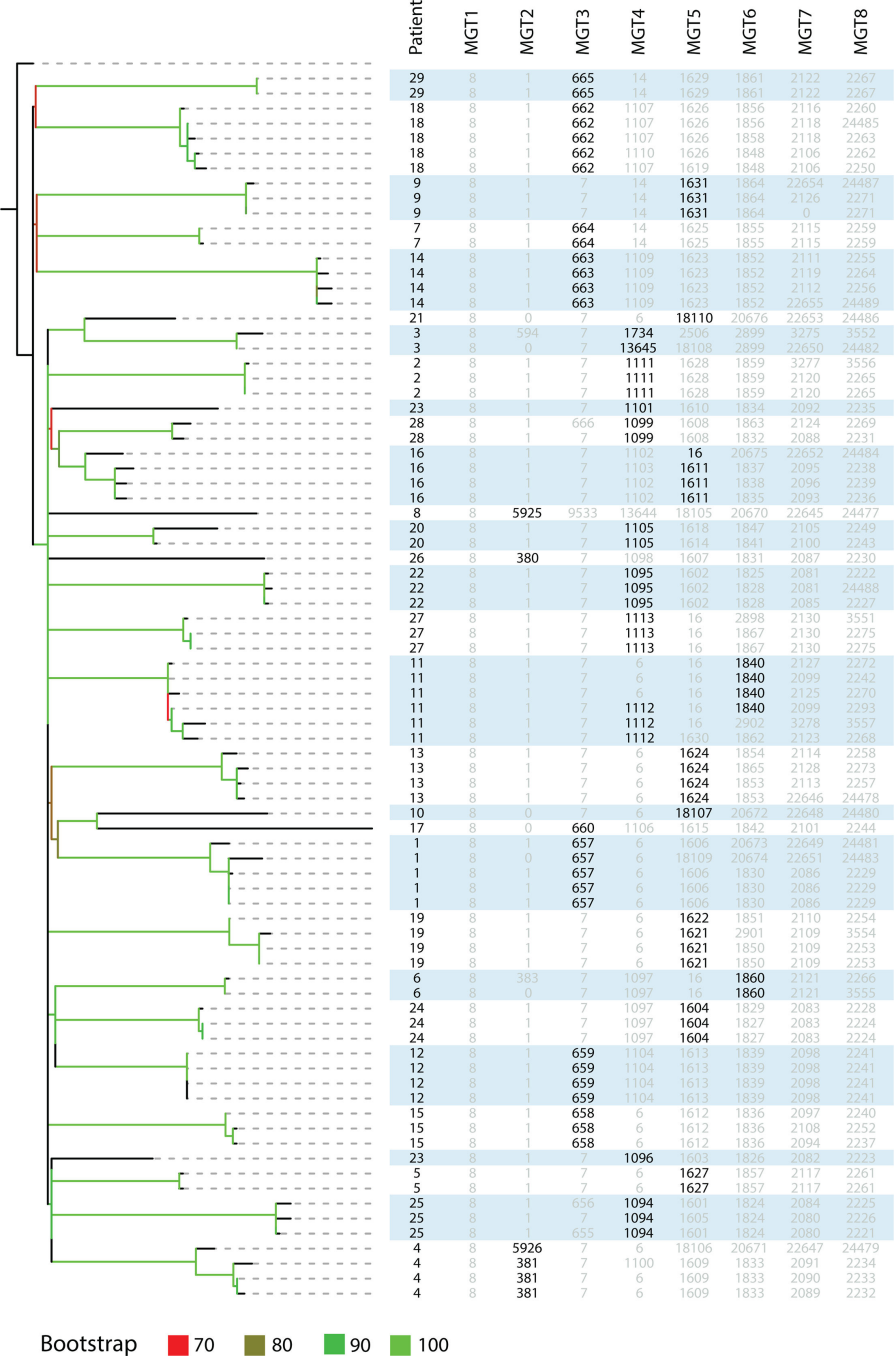
MGT1–MGT8 classification of MGT1 ST8-USA300 isolates from the same patient. A collection of MGT1 ST8-USA300 isolates (*n* = 82) was sampled from 29 patients and classified using MGT. The MGT1–MGT8 STs and anonymized patient identifiers for each isolate were aligned next to the phylogeny. STs that were selected to group isolates from the same patient are bolded. The STs in gray were not used to describe multiple isolates from the same patient. A phylogenetic tree was generated using maximum likelihood based on variations in core SNPs. Branches are colored based on bootstrap support values per color legend. Branches with bootstraps <70 were not colored and remained black.

We used WGS data from a study that tracked *S. aureus* colonization of different body sites of the same patients (34) to demonstrate the usefulness of multiple-level resolution of MGT. The 82 *S. aureus* MGT1 ST8-USA300 isolates from a cohort of 29 patients were typed by MGT, 24 of which had more than one isolate. A maximum likelihood phylogeny was constructed using core genome SNPs (Fig. 5).

Using the levels of MGT2 to MGT6, a total of 34 STs at different MGT levels were identified that were specific to one of the 24 patients. In 18 patients (1, 2, 5–10, 12–15, 17–18, 20–22, and 24–29), all isolates were assigned a specific MGT ST (Fig. 5). The remaining six patients (3, 4, 11, 16, 19, and 23) each had isolates typed by two specific STs (Fig. 5). Only Patient 11 required STs from two different MGT levels to describe all isolates, with three isolates each of MGT4 ST1112 and MGT6 ST1840.

We further identified the number of patient-specific STs at each MGT level and the percentage of the total isolates assigned to them. The STs from MGT1 to MGT5 were unable to assign all isolates to STs that were specific to one patient (Table 3). MGT6 was the first level containing only patient-specific STs. At MGT6, 13 patients required two or three STs to group all isolates. Seven patients (4, 20, 22–24, and 27–28) each had two MGT6 STs, and six patients (1, 11, 13, 16, and 18–19) each had three MGT6 STs, which grouped all isolates.

**TABLE 3:** Determination of MGT level to group all isolates of the same patient

Level	Patients described	Patient-specific STs	Isolates in patient-specific STs (% of data set)^*a*^
MGT1	0	0	0
MGT2	5	6	9.76
MGT3	11	12	36.59
MGT4	17	21	53.66
MGT5	27	35	86.59
MGT6	29	49	100
MGT7	29	61	100
MGT8	29	69	100

^*a*^ For MGT1 to MGT8, the number of patients in which all isolates were assigned patient-specific STs was counted. An ST was patient-specific when 100% of the isolates assigned that ST were sampled from a single patient. At the MGT level, the total number of isolates in patient-specific STs was reported as a percentage of the ST8-USA300 data set (*n = *82).

### MGT application to investigation of MGT1 ST239 transmission in a hospital setting

**Fig 6:**
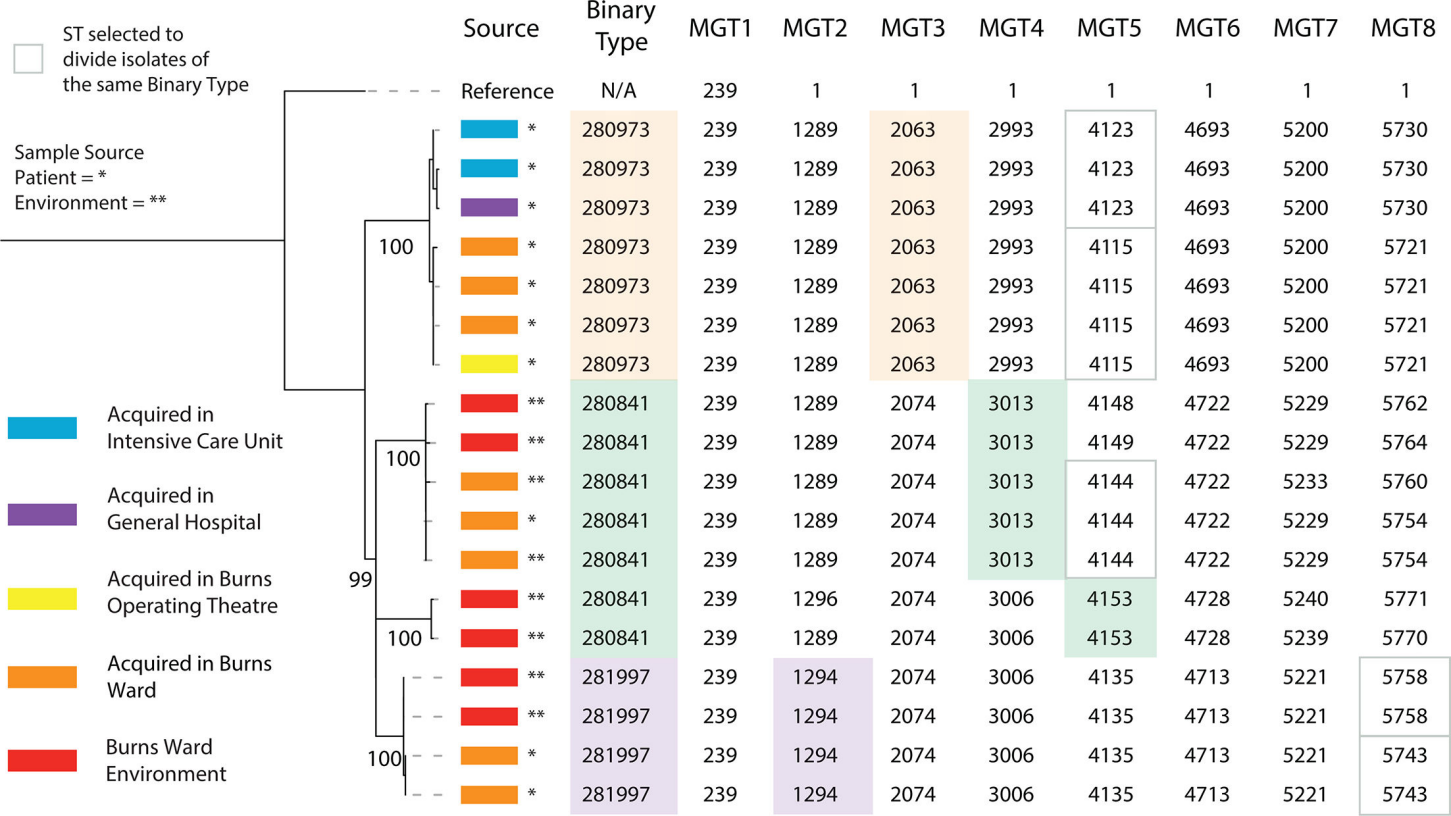
MGT classification of MGT1 ST239-SCC*mec* III spread within Concord Hospital. The spread of MGT1 ST239-SCC*mec* III through the Concord Hospital (Sydney, Australia) was described by MGT and binary typing. The three binary types are colored orange, green, and purple. The same coloring scheme was used to mark MGT STs that were selected to group isolates as binary types. MGT STs that divided binary types in concordance with the phylogenetic structure are outlined with a gray box. Patient-sampled isolates are marked with an asterisk, and isolates sampled from the environment are marked with a double asterisk.

In a published study that characterized the spread of MRSA throughout Concord Hospital (Sydney, NSW, Australia) between July 2012 and November 2014 (35), 238 MRSA isolates were collected and typed using multiplex PCR-reverse line blot binary typing (referred to as binary typing). Eighteen isolates were typed as (MGT1) ST239-SCC*mec* III and divided into three binary types (BTs): 280841, 280973, and 281997. When these isolates were typed using MGT, four MGT STs from MGT2 to MGT5 described the same sets of isolates as those described by BT (Fig. 6). MGT2 ST1294 and MGT3 ST2063 are equivalent to BT281997 and BT280973, respectively. MGT5 separated BT280973 into MGT5 STs (ST4115 and ST4123), which also represented isolates acquired from different hospital areas. MGT5 ST4115 was acquired within the burns operating theater and ward, whereas MGT5 ST4123 was acquired in the intensive care unit and general hospital area. Within the burns ward, BT281997 (MGT2 ST1294) was divided into MGT8 ST5758, which was patient-acquired, and MGT8 ST5743, which was from the hospital environment. Thus, MGT STs from MGT8 could distinguish patient isolates from those sampled from the environment. BT280841 was the only BT that could not be described by an ST at a single MGT level. BT280841 was separated into MGT4 ST3013 and MGT5 ST4153, with the latter containing only burn ward environment isolates. MGT4 ST3013 contained isolates sampled from patients and the environment, with the patient isolates distinguished by MGT5 ST4144.

## DISCUSSION

The development of genomic classification technologies that characterize *S. aureus* population structure and transmission is essential for designing and implementing control and prevention strategies. In this study, an MGT scheme was developed to classify all publicly available *S. aureus* WGS data (*n =* 50,481 as of 15 July 2019). The *S. aureus* MGT database is updated daily and publicly available for community use at https://mgtdb.unsw.edu.au/staphylococcus/. The public MGT database has minimal requirements for bioinformatics expertise. Sequencing reads can be directly uploaded to the server, which processes the data within a few hours and automatically assigns MGT STs at each of the eight MGT levels. These assignments and other data (such as alleles and allelic profiles) are made available to the user for further analysis. A comprehensive description of the usability and features of MGTdb, and its software architecture for local deployment, can be found in Kaur et al*.* (27). MGT was used to investigate both large- and small-scale *S. aureus* genomic epidemiology using published data as case studies.

### MGT for the standardized genomics-based classification of *S. aureus*

In 2007, the European Society of Clinical Microbiology and Infectious Diseases released guidelines for the development of novel classification technologies (45). These guidelines emphasize the importance of creating new typing technologies that define a standardized nomenclature, offer flexibility in typing resolution, and assign types that are interpretable and easily communicable. *S. aureus* MGT has eight levels filled with loci from the species core genome. The differing number of loci at different MGT levels offers flexibility in typing resolution, while maintaining standardized ST nomenclature at each level. These standardized MGT STs are easily interpretable to encourage standardized communication regarding *S. aureus* genomic epidemiology.

The range of typing resolutions makes the MGT a useful epidemiological tracing tool. At lower-resolution levels, STs tend to be larger, longer-lived clones and more widely distributed worldwide. As the resolution increases, STs become smaller, more short-lived, and continent- or country-specific. At the highest resolution, MGT8 (cgMLST) has the power to uncover chains of transmission and outbreak origins (20, 46, 47). The benefit of MGT classification is that when multiple levels are considered together, the higher-resolution level progressively divides the lower-resolution level STs to hierarchically reveal the relationship of the isolates across MGT levels. However, hierarchical inconsistencies may be present for a small proportion of the isolates across MGT1 to MGT7 because of the mutually exclusive loci sets that make up the MGT levels (22). Hierarchical inconsistency is also further illustrated in Fig. S4. Random mutations of genes at a lower-resolution level will lead to the assignment of an isolate to a different ST from its closest isolate, while at a higher-resolution level, the same ST will be assigned due to no mutations in genes at the higher-resolution level. This inconsistency can be resolved by examining the levels above and below to identify their true relationships. Further examination of clonal complexing of the involved STs can also help to resolve the inconsistencies.

We used several MGT levels above MGT1 (seven-gene MLST) to showcase the flexibility and usefulness of MGT to examine *S. aureus* population structure and epidemiological surveillance. In general, the lower-resolution levels such as MGT2–MGT5 can be used for longer-term epidemiology, while the higher-resolution levels such as MGT6–MGT8 can be used for shorter-term epidemiology. The estimates of the time of emergence of a new ST at a given MGT level are based on the average nucleotide substitution rate of 2.83 × 10^−6^ (48–51) (Fig. 1), which is also consistent with the average year span of the STs at different MGT levels from the global data set (Table 1; Fig. S1). Thus, the different MGT levels give an indication of their appropriateness for temporal epidemiological analysis ranging from >110 years when using MGT1 to 2 months when using MGT8. However, the selection of a specific MGT level or multiple levels will depend on the data and its epidemiological objectives.

We have shown that MGT offers high resolution across diverse data sets and could serve as a valuable tool for longitudinal studies that track the evolution and persistence of specific clones. Different MGT levels may be suited to examine *S. aureus* epidemiology in local, national, or international levels and to identify emerging clones in both hospital and community settings.

The highest level, MGT8, is species core genome MLST. Further typing resolution can be achieved by including core intergenic regions or constructing clone level core genome MLST. For *Salmonella* serovars Typhimurium and Enteritidis, a serovar level MGT9 increases resolution by 6%–18% based on the number of STs typed (22, 23).

### Multiple MGT levels describe global distribution of SCC*mec* types and PSSA isolates

The utility of describing populations at multiple levels using MGT was demonstrated using the global distribution of MRSA SCC*mec* types and PSSA strains. At MGT1, many STs are already composed of only one SCC*mec* type (ST22, ST105, and ST239); therefore, no higher resolution is required to describe their distribution. However, some globally prevalent MRSA STs can be divided into higher MGT-level STs that have either different SCC*mec* types or are MSSA. Within MGT1 ST5, MRSA types can be distinguished by MGT2 STs: SCC*mec* II by MGT2 ST1943, ST6485, ST6650, SCC*mec* IV by MGT2 ST3627, and MSSA by MGT2 ST1159. For MGT1 ST8, MSSA subtypes can be distinguished from MRSA isolates using MGT2 (ST199 and ST3854) and MGT3 (ST338).

Among the largest 100 MGT4 STs, PSSA is more sporadic and requires a higher resolution to separate it from non-PSSA isolates. Of the six MGT4 STs, two could also be uniquely identified by the lowest-resolution level at MGT1, two could be identified using MGT2, and the remaining two required MGT4 to uniquely identify them. Four of the PSSA MGT4 STs (ST594, ST885, ST1114, ST10735) were mostly composed of isolates from artificial evolution experiments (52, 53) and were all within CC8, whereas ST14648 and ST9209 were from studies on avian *S. aureus* and general *S. aureus* diversity, respectively, and were from CC5 (54, 55).

### MGT provides better description of the large-scale population structure of globally disseminated clones

MGT1 ST8 is a global CA-MRSA ST that has spread in the Americas and Europe, but has also been reported in Africa and Asia (13, 56–58). Using MGT2, we can describe country-specific STs for the USA (MGT2 ST114, ST199, and ST443) and the UK (MGT2 ST146). In contrast, we can also identify STs that are still globally distributed, even at a significantly higher resolution of MGT4 (MGT4 ST6 and ST14). Within MGT1 ST8, the USA300 clone is of particular interest because of its hypervirulence and its association with *spa* type t008 and SCC*mec* type IV. MGT2 ST1 was the dominant MGT2 ST within MGT1 ST8 and was dominated by SCC*mec* type IV and *spa* type t008 isolates. MGT2 STs also describe other large *spa* types, such as t064 (MGT2 ST114), which is associated with Africa (59), and t211 (MGT2 ST199). MGT provides simpler descriptions of many MGT1 ST8 clades than *spa* types. Several *spa* types that are polyphyletic are divided into smaller but more phylogenetically congruent MGT2 STs, such as *spa* t064, which is divided into MGT2 ST114 and ST184. In addition, many small *spa* types can be more easily grouped into larger types using MGT2 STs. MGT can dissect the spatial population structure of global clones and provides a better description of the large-scale population structure than SCC*mec* typing and *spa* typing for MGT1 ST8.

### MGT STs can uniquely describe isolates colonizing the same patient within ST8-USA300

The presence of *S. aureus* is an important risk factor when predicting MRSA onset, and 50%–80% of MRSA infections are caused by isolates already carried by the host (60). The ability of WGS-based classification to type isolates sampled from the same patient can describe the genomic epidemiology of persistently colonizing *S. aureus*. The flexibility in MGT typing resolution allows STs from multiple levels to describe the persistent colonization of patients. As shown by the MGT1 ST8-USA300 isolates, STs from MGT2 to MGT6 were able to uniquely group MGT1 ST8-USA300 isolates of most patients by using a specific MGT typing resolution, with the majority of patients (24/29) having one unique MGT ST identifier. A combination of two or more levels would allow description of the origin and diversity within a patient.

### MGT STs described MGT1 ST239- SCC*mec* III spread throughout Concord Hospital, Sydney

MGT was used to describe the spread of MGT1 ST239-SCC*mec* III isolates in a hospital in a previous study (35). The multiple resolutions were able to describe both the spread between wards and identify isolates that colonized patients or environments within a ward. The STs from higher MGT levels separated isolates that colonized the hospital environment from the patients admitted to that ward, showing that higher MGT resolutions can separate MGT1 ST239-SCC*mec* isolates independently evolving within a hospital ward. Compared with binary typing, the flexibility in MGT typing resolution divides binary types into STs that better describe *S. aureus* acquisition and transmission. Higher resolution can distinguish more closely related isolates within a hospital ward, such as those colonizing a hospital environment and those colonizing an admitted patient. The application of MGT to describe MGT1 ST239-SCC*mec* III spread within a hospital acts as a proof-of-concept investigation and can be applied to any *S. aureus* control within a healthcare setting.

### Limitations of this study and MGT classification of *S. aureus*

Investigating *S. aureus* genomic epidemiology in this study used publicly available *S. aureus* WGS data and metadata. The availability of accurate metadata is essential for interpreting *S. aureus* WGS analysis. Almost all temporal metadata for the investigation of MGT1 ST8 large-scale population structure were sourced from NCBI BioSamples. Interpretation of *S. aureus* WGS data for temporal or spatial epidemiology is also hampered when metadata is not available. Only 50% of the data set had both year and country metadata. Improving metadata availability in the future will greatly enhance the genome data for a detailed description of species-wide *S. aureus* genomic epidemiology and global surveillance. The agreement on international standards for epidemiological metadata, such as country of origin, year of isolation, and host and disease-causing status, would enable better use of MGT to describe the genomic epidemiology of *S. aureus*.

A technical challenge when using MGT to describe *S. aureus* genomic epidemiology is the definition of singleton STs. The division of *S. aureus* genomes into singletons prevents a description of the genetic relationship between the isolates. Higher MGT levels that offer higher resolutions are expected to increasingly separate isolates into singletons. For example, the MGT2 classification of MGT1 ST8 defined 9.80% (430/4,388) of the isolates as singleton STs, which were excluded from further investigation. It is possible that the isolates in these singleton STs were closely related to those in one of the other large MGT2 STs. The MGT2 level had 21 core loci, and an isolate assigned to a singleton ST needed to carry only a unique allele for one of the 21 loci. These singleton STs, likely representing novel variants, have previously been shown to arise more frequently in bacterial species with higher SNP mutation rates such as *S. aureus* (61). In traditional MLST schemes, CC is used to group singleton STs with their member of generally one or two allele difference (31, 62), which is widely used in the *S. aureus* MLST (63). In the *S. aureus* MGT online database, clonal complexes (defined by one allele difference between member STs) are automatically generated at each MGT level to enable users to view the clonal complex of singleton STs at each MGT level. Additionally, in general, singleton STs do not limit resolution in transmission inference during outbreak tracking, as they are often incorporated into outbreak clusters. Outbreak delineation typically relies on genomic distance cutoffs (SNPs or alleles) and contextual information such as timeframe, geography, or within-host diversity (64, 65). Thus, if a singleton ST differs from outbreak-associated strains by only a single SNP/allele, or by a number of SNPs/alleles below the defined threshold, then that ST is generally included within the outbreak cluster.

### Future directions and public health implications

We show in this study that MGT can be used for standardized genomic surveillance of *S. aureus*. It has the potential for real-time public health surveillance with or without integration into judiciary-specific public health surveillance systems. We have developed basic surveillance reports that are generic to all MGT schemes (27) and can be further developed to cater to specific organisms. A major barrier to the application of genome data to public health is bioinformatic analysis, which often involves phylogenetic analysis and arbitrary SNP cutoffs (64). The turnaround time for bioinformatic analysis in an infection control application study was 6 days on average (65). The turnaround of MGT typing is short (generally approximating 4 to 6 hours), which is largely automated. This is particularly useful in settings with limited bioinformatics resources. We previously developed an outbreak detection algorithm for foodborne pathogens (66). It can be further developed for *S. aureus* and integrated into the *S. aureus* MGT system. It may further be used in conjunction with specialist infection control tools such as HAIviz (67), which has the capability for geotagging within a facility. Therefore, MGT can be used to monitor potential outbreaks in healthcare settings and support outbreak response, for instance, by guiding interventions within specific hospital wards or among specific patient groups.

*S. aureus* MGT can be further integrated with antimicrobial resistance gene profiling and virulence gene profiling to detect and track existing or emerging antimicrobial-resistant or virulent clones (68). *S. aureus* MGT can also be integrated with artificial intelligence to predict transmission pathways based on genome data in the future. Lastly, future studies can also aim to integrate genomic data with phenotypic data, such as antimicrobial resistance, virulence, and environmental persistence traits, as this will strengthen the translational relevance of genomic surveillance.

### Conclusion

In this study, we developed an MGT scheme for flexible, stable, and standardized genomic classification of *S. aureus*. The MGT consists of eight levels, providing typing resolution flexibility. MGT describes the large-scale population structure of the species, including global, continent-specific, and country-specific STs, and their relationship to MRSA lineages and SCC*mec* subtypes. Within the globally disseminated MGT1 ST8, MGT2 was able to match or improve upon the commonly used *spa* typing method. Using a combination of higher-resolution MGT levels, MGT STs were able to precisely describe isolates from each patient from a study of persistent colonization. Finally, in a hospital outbreak, MGT STs from lower levels grouped isolates that had spread between hospital wards, whereas higher MGT levels assigned STs that distinguished isolates within the same ward. The MGT was able to describe the genomic epidemiology of *S. aureus* from large, long-lived, global STs to small, short-lived STs found only in a single patient. The *S. aureus* MGT is publicly available (https://mgtdb.unsw.edu.au/staphylococcus/), updated daily, and allows both public and private user submission. *S. aureus* MGT can assist in the tracking of existing and new *S. aureus* clones, which is essential when designing prevention and control strategies to reduce the disease burden of this important pathogen.

## Data Availability

All data used in this study are publicly available, and all MGT data are available at mgtdb.unsw.edu.au/Staphylococcus.

## SUPPLEMENTAL MATERIAL

Figure S1 msystems.00935-25-s0001.pdf Distribution of year spans of major STs at each MGT level.

Figure S2 msystems.00935-25-s0002.pdf The size distribution of MGT2 to MGT8 STs assigned to MGT1 ST8 isolates.

Figure S3 msystems.00935-25-s0003.pdf Comparing MLST ST8 classification between MGT and Spa typing.

Figure S4 msystems.00935-25-s0004.pdf Illustration of hierarchical inconsistency at MGT3 in six isolates.

Supplemental text msystems.00935-25-s0005.docx Supplemental methods.

Supplemental data sets msystems.00935-25-s0006.xlsx Data sets S1 to S9.

Table S1 msystems.00935-25-s0007.xlsx Criteria for quality-filtering assemblies.

Table S2 msystems.00935-25-s0008.txt The quality-filtered species data set with MGT assignment and available metadata.

ASM does not own the copyrights to Supplemental Material that may be linked to, or accessed through, an article. The authors have granted ASM a non-exclusive, world-wide license to publish the Supplemental Material files. Please contact the corresponding author directly for reuse.
