# Supplementary material for: Characterizing Staphylococcus aureus genomic epidemiology with multilevel genome typing
Source: mSystems. 2025 Oct 2;10(10):e00935-25. doi: 10.1128/msystems.00935-25 (PMC12542621; doi:10.1128/msystems.00935-25)
Supplement: Figure S1 — Distribution of year spans of major STs at each MGT level. [file msystems.00935-25-s0001.pdf]

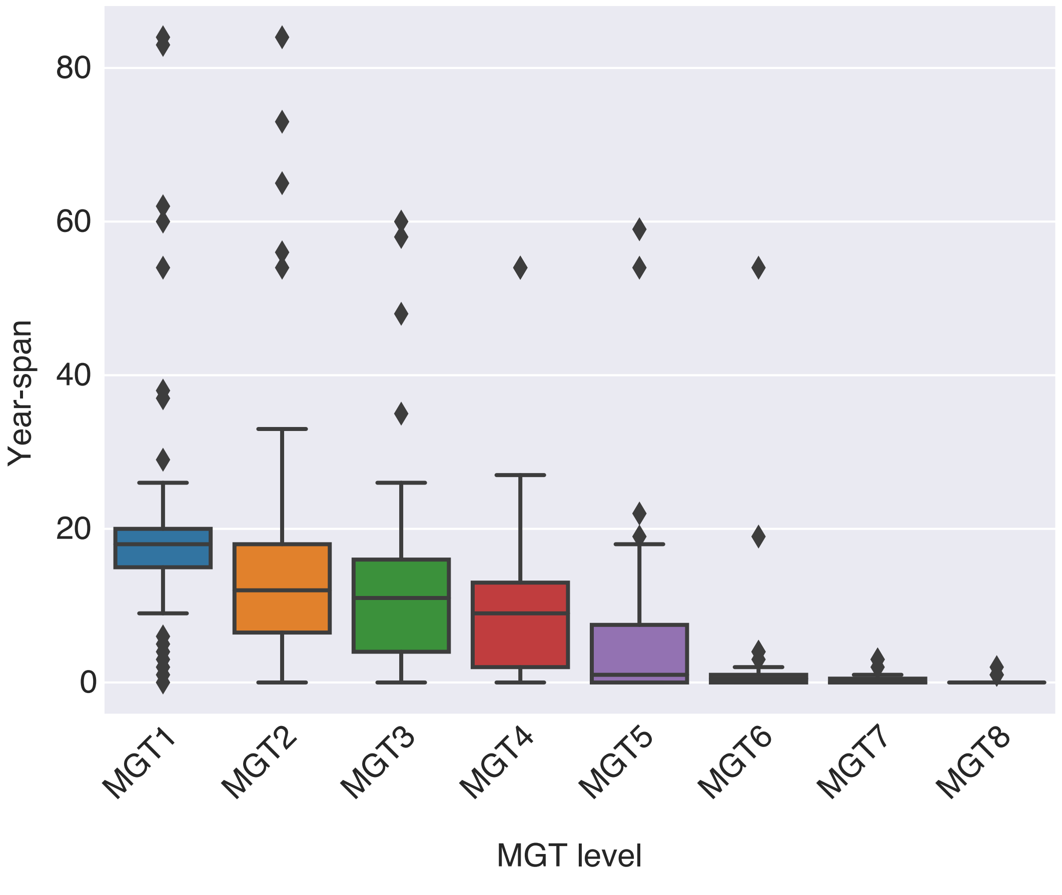

Supplementary Figure 1. Distribution of yearspans of major STs at each MGT level. For each MGT level the year-span of every ST with more than 10 isolates assigned to it (major ST) was calculated and their distribution is plotted on the Y-axis. The X axis is each of the 8 *S. aureus* MGT levels
