# Supplementary material for: Characterizing Staphylococcus aureus genomic epidemiology with multilevel genome typing
Source: mSystems. 2025 Oct 2;10(10):e00935-25. doi: 10.1128/msystems.00935-25 (PMC12542621; doi:10.1128/msystems.00935-25)
Supplement: Figure S2 — The size distribution of MGT2 to MGT8 STs assigned to MGT1 ST8 isolates. [file msystems.00935-25-s0002.pdf]

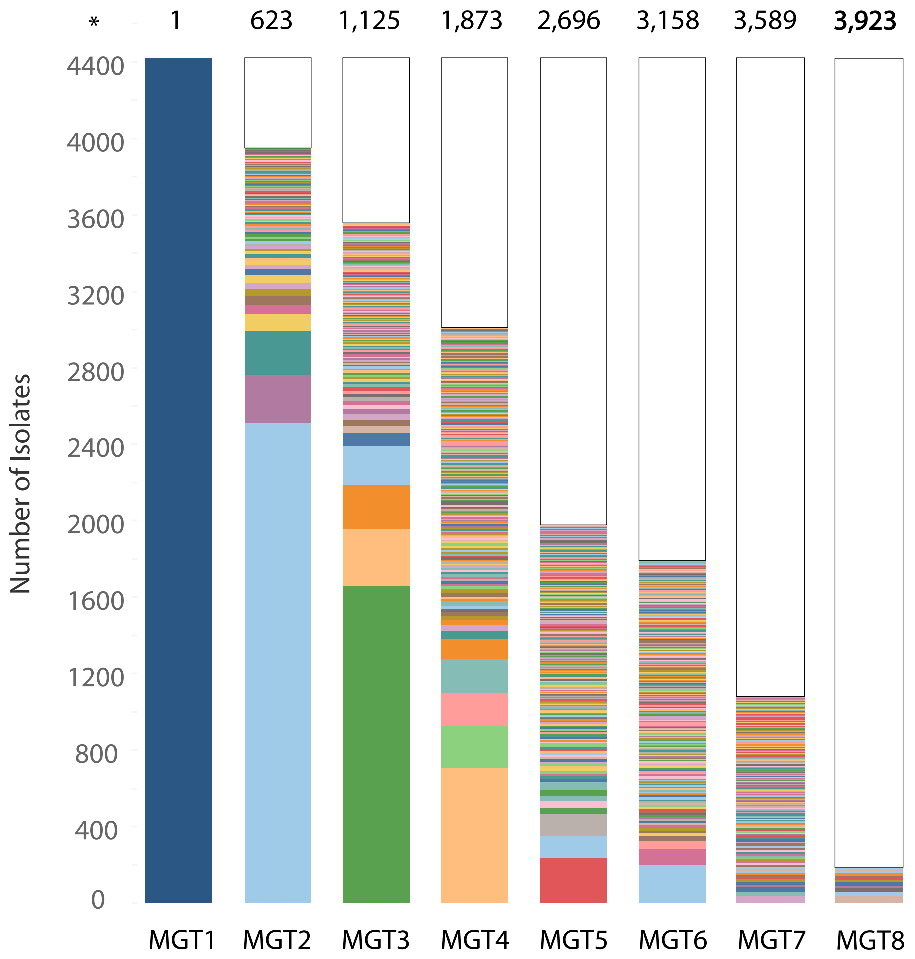

Supplementary figure 2. The size distribution of MGT2 - MGT8 STs assigned to MGT1 ST8 isolates.
The MGT was used to classify isolates in the MGT1 (MLST) ST8 dataset (n=4,388). The sizes of STs are represented as coloured bars at each MGT level. STs that are assigned to a single isolate (singleton) were collapsed into a single white box. The total number of STs assigned at an MGT level are labelled above (marked by asterisk).
