# Supplementary material for: Characterizing Staphylococcus aureus genomic epidemiology with multilevel genome typing
Source: mSystems. 2025 Oct 2;10(10):e00935-25. doi: 10.1128/msystems.00935-25 (PMC12542621; doi:10.1128/msystems.00935-25)
Supplement: Figure S4 — Illustration of hierarchical inconsistency at MGT3 in six isolates. [file msystems.00935-25-s0004.pdf]

a. Sequence types

Isolate	Server status	Assignment status	MGT1 ST	MGT2 ST	MGT3 ST	MGT4 ST	MGT5 ST	MGT6 ST	MGT7 ST	MGT8 ST
SRR1050103	Complete	Assigned MGT	8	1	7	6	655	731	784	812
ERR957900	Complete	Assigned MGT	8	1	7	6	655	731	784	812
ERR235039	Complete	Assigned MGT	8	1	7	6	494	549	581	603
SRR1145884	Complete	Assigned MGT	8	1	7	6	494	549	581	603
SRR1050123	Complete	Assigned MGT	8	1	176	6	468	517	548	569
ERR234987	Complete	Assigned MGT	8	1	176	6	468	517	548	569

b. Clonal complexes

Isolate	Server status	Assignment status	MGT1 ST	MGT2 CC	MGT3 CC	MGT4 CC	MGT5 CC	MGT6 CC	MGT7 CC	MGT8 CC
SRR1050103	Complete	Assigned MGT	8	1	4	6	473	672	748	770
ERR957900	Complete	Assigned MGT	8	1	4	6	473	672	748	770
ERR235039	Complete	Assigned MGT	8	1	4	6	357	504	555	571
SRR1145884	Complete	Assigned MGT	8	1	4	6	357	504	555	571
SRR1050123	Complete	Assigned MGT	8	1	4	6	7	476	523	538
ERR234987	Complete	Assigned MGT	8	1	4	6	7	476	523	538

Supplementary Figure 4. Illustration of hierarchical inconsistency at MGT3 in six isolates. Hierarchical inconsistency occurs when a lower MGT level indicates that a given set of isolates are distantly related due to different ST assignments at that level, while the upper MGT level indicates that they are closely related due to the same ST assignment at this level. a) Six isolates are hierarchically inconsistent at MGT3 (outlined in red), as the first four isolates are assigned MGT3 ST7, while the bottom two isolates are assigned MGT3 ST176. All isolates are assigned ST6 at the MGT4 level (the higher resolution level). Hierarchical inconsistency arises due to mutually exclusive sets of loci used in the schemes of the different MGT levels. Mutations can arise at any loci (including at a lower level), resulting in a new ST. Hierarchical inconsistency makes it difficult to infer relationships between isolates using a single level. However, b) shows that all six isolates at MGT3 are within the same single linkage cluster (or clonal complex, CC) MGT3 CC4, indicating the close relationship of the six isolates.
