# Supplementary material for: Characterizing Staphylococcus aureus genomic epidemiology with multilevel genome typing
Source: mSystems. 2025 Oct 2;10(10):e00935-25. doi: 10.1128/msystems.00935-25 (PMC12542621; doi:10.1128/msystems.00935-25)
Supplement: Supplemental text — Supplemental methods. [file msystems.00935-25-s0005.docx]

**Supplementary Methods**

**1.1 Calculating the size of each level**

The number of loci per MGT2-7 level was calculated using a whole genome SNP mutation rate and selecting the amount of DNA expected to generate a single SNP in fixed timeframes (**Supplementary Dataset 1, Supplementary Methods Table 1**) [1-4].

**Supplementary Methods Table 1.** Target time periods for each MGT level and the corresponding basepair length needed to achieve one mutation in that time period.

| **Level** | **Level Time Period (Years)** | **Target Size of Level (basepairs)** |
| --- | --- | --- |
| MGT2 | 20 | 17,559 |
| MGT3 | 10 | 35,118 |
| MGT4 | 5 | 70,236 |
| MGT5 | 2 | 175,589 |
| MGT6 | 1 | 351,178 |
| MGT7 | 0.5 | 702,356 |

**1.2 Calculating locus filtering metrics**

The following metrics were then calculated for each locus of the core genome: dN/dS ratio, locus quality, homopolymers, tandem repeats and phage coding regions (**Supplementary Datasets 2 to 6**). Calculated metrics unique to this study were a rate of allelic change per kilobase and a predicted number of recombination events per locus (**Supplementary Datasets 7 and 8, Supplementary Scripts 1 and 2**).

**1.3 Calculating the rate of allelic change per kilobase**

The rate of allelic change for a core locus was used as a proxy for measuring the rate of evolution. The rate represented the number of alleles per kilobase for a locus. Per core locus, the rate of allelic change was the average of two test datasets each with 10,000 genomes. Each dataset represented the seven-gene MLST diversity of species. An in-house python script sampled each ST proportionally based on that STs frequency in the species dataset (**Supplementary Script 1**). A single isolate was selected from all singleton STs. Allele profiles for the test datasets were generated using the MGTdb Allele Calling pipeline [5]. Prism was used to compare the distribution of alleles per core gene. The means of test-dataset one and test-dataset two were compared with a Mann-Whitney U test. Significance between means was interpreted with an alpha threshold of 0.95. The number of alleles per core gene was averaged between the two datasets and normalised per kilobase.

Core loci were separated into percentiles based on the distribution of rate of allelic change per kilobase. The distribution of values was tested for normality using a Shapiro-Wilk test. A *p*-value less than or greater 0.05 and alpha value higher than 20 indicate a distribution departing from normality. Genes were categorised into the following percentiles within a negative binomial distribution: 10th, 20th, 30th, 40th, 50th, 60th, 70th, 80th, 90th and 100th.

**1.4 Calculating the predicted number of recombination events per locus**

The number of recombination events impacting each of the core loci was predicted. A core SNP alignment was generated for representative dataset using Snippy [6]. Isolates of the representative dataset were aligned to *S. aureus* COL. The regions predicted to be under the influence of recombination were identified with RecDetect [7]. A strict prediction model for high recombination species was selected. For each core locus, the number of recombinant regions that overlapped was counted with an in-house python script (**Supplementary Script 2**). Both partially and completely overlapping regions were identified. A partial overlap was a recombinant region that overlapped the start or end positions of a locus. A complete overlap was a recombinant region within the start and end positions of a locus. The number of recombination events per core locus was visualised in Prism [8].

**1.5 Separating the core loci into preferences**

All calculated metrics were the basis for separating the core loci with a preference-based system [5,9,10]. An in-house python script separated the core loci into preferences 1- 12 using the filtering thresholds in **Supplementary Methods Table 2 (Supplementary Script 3)**. MGT2-7 were filled by randomly selecting loci from the lowest preference. The levels were filled in ascending order with loci that were separated by the distances shown in **Supplementary Methods Table 3**. The loci from the next highest preference were selected when an MGT level required additional loci to meet the scheme size **(Supplementary Script 3)**.

**1.6 Mutually exclusive locus assignment and hierarchical inconsistency**

Loci in MGT levels 2 to 7 are mutually exclusive which allows for independent characterisation of population structure at each level. This independence also means that an ST at a higher resolution level (i.e. MGT4) can be classified into multiple STs at a lower resolution (i.e. MGT3), this is known as hierarchical inconsistency and has been described in detail previously [5].

**1.7 Core genome quality filtering methods**

The loci of core genome defined in 2014 were verified [11]. Alleles for each locus were called with the Allele Calling Pipeline from the MultiLevel Genome Typer [5]. Core genes were validated with isolates from the representative dataset. The 1,861 loci were called using the Allele Calling Pipeline from the MultiLevel Genome Typer. Selected settings were a BLAST (v2.9) similarity of 80% and a 16 SNP sliding window.

**Supplementary Methods Table 2.** Preference classifications assigned to each locus depending on their characteristics

| **Preference** | **Allelic Rate Percentile** | **dN/dS Ratio Percentile** | **Missing (%)** | **Problematic Allele Calling (%)** | **Recombination Events** | **Homopolymer** | **Tandem Repeats** | **Phage Region** |
| --- | --- | --- | --- | --- | --- | --- | --- | --- |
| 1 | 10 | 30 | <=1 | <=1 | <=15 | False | False | False |
| 2 | 20 |  |  |  |  |  |  |  |
| 3 | 30 |  |  |  |  |  |  |  |
| 4 | 40 | 50 |  |  | <=20 |  |  |  |
| 5 | 50 |  |  |  |  |  |  |  |
| 6 | 60 | 90 |  |  |  |  |  |  |
| 7 |  |  |  |  |  |  |  |  |
| 8 | 70 |  |  |  | <=25 |  |  |  |
| 9 | 90 |  |  |  |  |  |  |  |
| 10 | 95 | 100 |  |  |  |  |  |  |
| 11 | 100 |  |  |  |  |  |  |  |
| 12 |  |  |  |  |  | True | True | True |

Note: The core loci were divided into preferences 1-12. Multiple metrics for each core locus were considered when assigning a preference. The filtering thresholds used to rank loci into preferences were shown.

**Supplementary Methods Table 3.** Minimum allowable distance between isolates.

| **MGT level** | **Separation Between Loci (bases)** |
| --- | --- |
| MGT2 | 20,000 |
| MGT3 | 12,500 |
| MGT4 | 5,000 |
| MGT5 | 3,500 |
| MGT6 | 500 |
| MGT7 | 0 |

**References**

[1] Harris SR, Feil EJ, Holden MT, et al. Evolution of MRSA during hospital transmission and intercontinental spread. Science. 2010;327(5964):469-474.

[2] Smyth DS, McDougal LK, Gran FW, et al. Population structure of a hybrid clonal group of methicillin-resistant *Staphylococcus aureus*, ST239-MRSA-III. PLoS One. 2010;5(1):e8582.

[3] Young BC, Golubchik T, Batty EM, et al. Evolutionary dynamics of *Staphylococcus aureus* during progression from carriage to disease. Proceedings of the National Academy of Sciences. 2012;109(12):4550-4555.

[4] Nübel U, Dordel J, Kurt K, et al. A timescale for evolution, population expansion, and spatial spread of an emerging clone of methicillin-resistant *Staphylococcus aureus*. PLoS pathogens. 2010;6(4):e1000855.

[5] Payne M, Kaur S, Wang Q, et al. Multilevel genome typing: genomics-guided scalable resolution typing of microbial pathogens. Euro Surveill. 2020 May;25(20):1900519.

[6] Seemann T. Snippy: Rapid haploid variant calling and core genome alignment. 2015. Available from: <https://github.com/tseemann/snippy>

[7] Hu D, Liu B, Wang L, et al. Living Trees: high-quality reproducible and reusable construction of bacterial phylogenetic trees. Molecular Biology and Evolution. 2019 Feb 1;37(2):563-575.

[8] Hart EM, Bell K. prism: Download data from the Oregon prism project. R package version 00. 2015;6(10.5281).

[9] Luo L, Payne M, Kaur S, et al. Elucidation of global and national genomic epidemiology of *Salmonella enterica* serovar Enteritidis through multilevel genome typing. Microb Genom. 2021 Jul;7(7):000605.

[10] Cheney L, Payne M, Kaur S, et al. Multilevel Genome Typing Describes Short- and Long-Term *Vibrio cholerae* Molecular Epidemiology. mSystems. 2021 Aug 31;6(4):e0013421.

[11] Leopold SR, Goering RV, Witten A, et al. Bacterial whole-genome sequencing revisited: portable, scalable, and standardized analysis for typing and detection of virulence and antibiotic resistance genes. Journal of Clinical Microbiology. 2014 Jul;52(7):2365-70.
